# Germ Cell Tumor Complicated by Mediastinal Mass Syndrome: A Report of Cardiac Arrest to Full Recovery

**DOI:** 10.7759/cureus.46445

**Published:** 2023-10-03

**Authors:** Razan Odeh, Mo’tasem Dweekat, Ali Shakhshir

**Affiliations:** 1 Department of Hemato-Oncology, An-Najah National University Hospital, Nablus, PSE; 2 Department of Internal Medicine, An-Najah National University Hospital, Nablus, PSE; 3 Department of Internal Medicine, Al Watani Hospital, Ministry of Health, Nablus, PSE

**Keywords:** mediastinal mass syndrome, post-intubation complications, cardiac arrest, anterior mediastinal mass, mediastinal germ cell tumor

## Abstract

Germ cell tumors (GCTs) are the most common malignancies in men aged 15-35 years. Five percent of malignant GCTs are of extragonadal origin, and the most common extragonadal location for GCTs in adults is the mediastinum. Like other mediastinal tumors, mediastinal GCTs may cause compression or invasion of vital mediastinal structures, resulting in respiratory or hemodynamic compromise. Right ventricular failure following positive pressure ventilation of such patients is called mediastinal mass syndrome (MMS). This report presents a case of a GCT complicated by cardiac arrest shortly after starting positive pressure ventilation, which was successfully resuscitated. Few previous reports demonstrated a successful outcome of MMS. This report highlights the importance of a multidisciplinary approach for such scenarios in light of the scanty literature and lack of clear guidance and the significance of starting chemotherapy in a timely manner.

## Introduction

Mediastinal syndromes are a group of disorders characterized by variable presentations depending on the anatomic location and the specific structures involved. Pathologies leading to this syndrome can be divided into malignant and non-malignant types. They can also be divided according to the location of origin of the mass: anterior, middle, and posterior mediastinal. Regardless of the location and the cause, the consequences of these syndromes are caused by compression or entrapment of mediastinal structures within a confined space.

Superior vena cava (SVC) syndrome is said to be the most devastating consequence of mediastinal malignancies [1]. It is a collection of signs and symptoms mainly caused by partial or complete SVC obstruction. This obstruction leads to impaired venous return and venous congestion. The reported median survival of patients with malignant SVC syndrome is six months [2].

In adults, the mediastinum is the most common extragonadal location for germ cell tumors (GCTs). GCTs are usually tumors of the gonads and mainly arise in the adolescent and young adult age groups. Malignant GCTs are divided into seminomatous and non-seminomatous tumors. According to the International Germ Cell Cancer Collaborative Group (IGCCCG), primary mediastinal non-seminomatous GCTs are considered poor prognostic tumors [3]. The mainstay of therapy for such tumors is cisplatin-based chemotherapy and surgical resection [4].

Herein, we present a case of a primary mediastinal GCT complicated by cardiac arrest due to mediastinal mass syndrome (MMS), protracted intensive care unit care, and finally successfully managed with chemotherapy and mass resection.

## Case presentation

**Figure 1:**
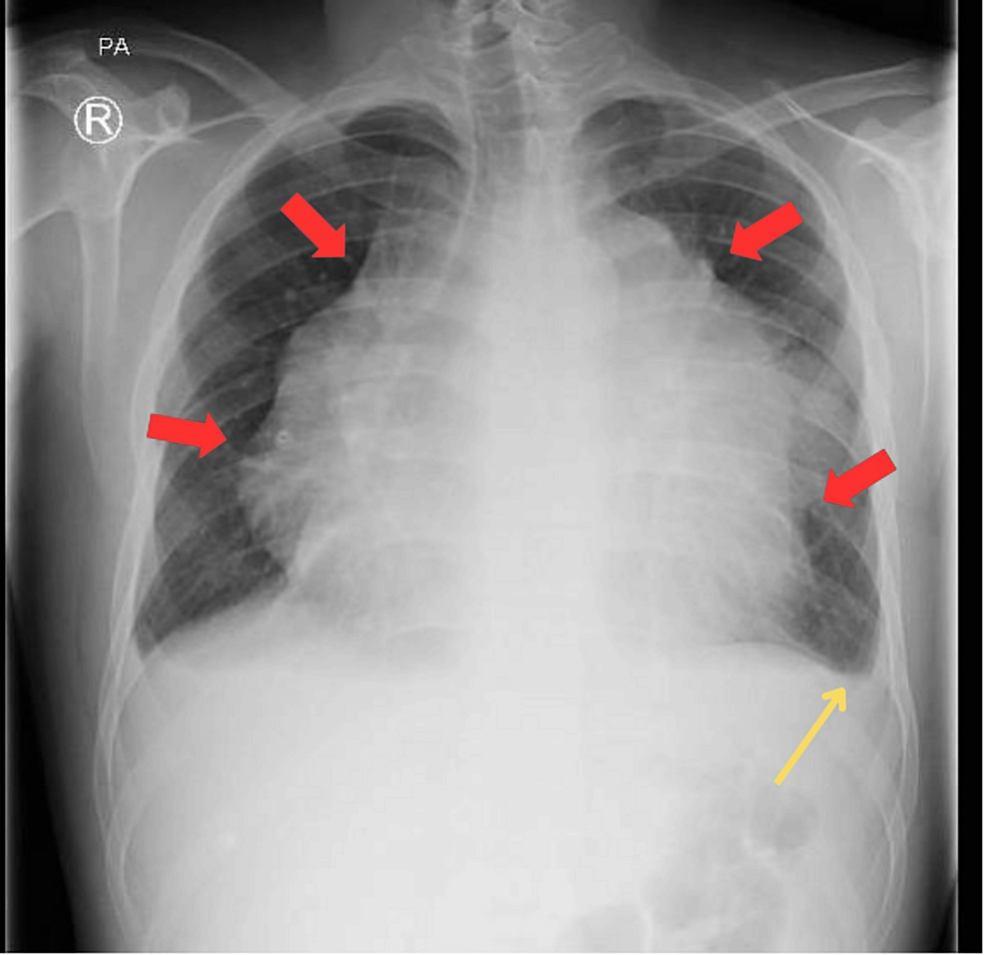
A posterior-anterior chest X-ray with red arrows surrounding the large mediastinal mass overlying the left lung field. Note the obliteration of the left costophrenic angle, which is pointed out by the yellow arrow.

A 22-year-old male with a free past medical history complained of pleuritic retrosternal chest pain associated with orthopnea, exertional dyspnea, and a dry cough in July 2020. He sought advice in an outpatient clinic, where he underwent basic investigations, including a CBC, which showed microcytic hypochromic anemia, and a chest radiograph, which revealed a huge mediastinal mass as shown in Figure 1, for which he was referred for evaluation.

**Figure 2:**
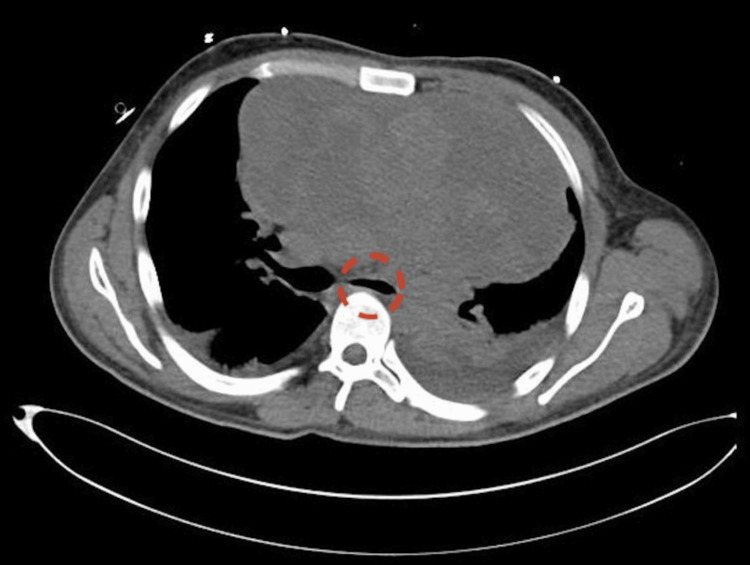
A CT scan of the thorax shows the mediastinal mass with significant compression of the patent left mainstem bronchus (dashed circle).

A computed tomography (CT) scan of the chest showed an anterior mediastinal heterogeneous soft tissue mass with a heterogeneous enhancement of 19 cm in maximal diameter, causing narrowing of the left mainstem bronchus, as shown in Figure 2, and the left innominate vein, shifting the mediastinum, compressing the patent pulmonary arteries, and a pericardial effusion of 3.2 centimeters in maximal thickness.

**Figure 3:**
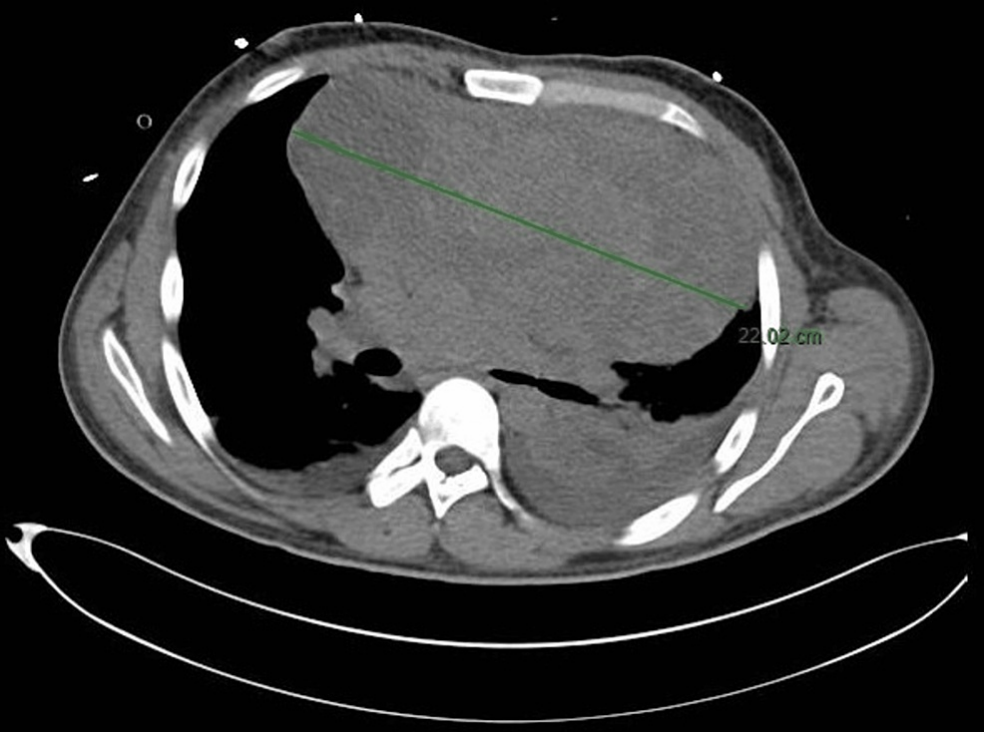
A CT scan of the thorax shows an anterior mediastinal mass of 22 cm in largest diameter. It is causing significant compression of the left lung.

At the time, alpha-fetoprotein was 2000 IU/mL (reference range: 0-5.8 IU/mL), beta-human chorionic gonadotropin (beta-hCG) was 140 mIU/mL (reference range for males: <2 mIU/mL), and lactate dehydrogenase (LDH) was 492 U/L (reference range: 45-245 U/L). Otherwise, except for microcytic hypochromic anemia with a hematocrit of 33.3% (reference range: 40-52%), his complete blood count, complete metabolic panel, and coagulation profile were unremarkable. He then underwent a CT-guided biopsy of the mass and was referred to our hospital. On presentation, his blood pressure was 119/65, his heart rate was 120, his oxygen saturation was 97% on a two-liter nasal cannula, and his respiratory rate was 18-20. His examination was remarkable for distended neck veins and muffled heart sounds, but otherwise, it was unremarkable. Transthoracic echocardiography showed a large circumferential pericardial effusion with Doppler evidence of cardiac tamponade, so he underwent pericardiocentesis with drainage of 250 cc of serous fluid, after which he was transferred to the surgical intensive care unit (SICU). His echocardiography also showed a dilated right ventricle, tricuspid valve regurgitation, and a high pulmonary artery systolic pressure (PASP) of 75 mmHg. A few days into admission, the patient developed a spiking fever, increasing shortness of breath, cough, and desaturation consistent with hospital-acquired pneumonia and was treated with intravenous antibiotics. By that time, histopathology for the mediastinal mass confirmed a non-seminomatous GCT. At that time, tumor markers were an alpha-fetoprotein of 46585 IU/mL, beta-hCG of 193 mIU/mL, and LDH of 288 U/L. Once he was out of sepsis, he was started on the etoposide, ifosfamide, and cisplatin (VIP) protocol. The testicular ultrasound was unremarkable. Over the next few days, the patient developed progressively increasing respiratory distress with increasing oxygen requirements with no improvement on non-invasive mechanical ventilation, and it was decided to proceed with intubation. Propofol, fentanyl, and succinylcholine were used for the induction of anesthesia. He developed hemodynamic instability, which was successfully managed with IV fluid boluses and low-dose vasopressors. Aspiration of pericardial fluid yielded 20 cc with no improvement. This raised suspicion of pulmonary embolism, so he was sent for CT pulmonary angiography (CTPA) on low-dose vasopressors and stable respiratory requirements. He developed rapid hemodynamic and respiratory decompensation in the radiology department, followed closely by cardiac arrest with pulseless electrical activity. Cardiopulmonary resuscitation (CPR) for four minutes with 1 mg of intravenous epinephrine was followed by return of spontaneous circulation (ROSC). The CTPA revealed no evidence of pulmonary embolism but showed an increase in the mass size to 22 cm in maximal diameter, as shown in Figure 3, along with significant compression of nearby major vessels, near complete collapse of the left lower lung lobe, and bilateral pleural effusions.

Due to his respiratory condition, left-sided thoracentesis was done with drainage of 1300 cubic centimeters (cc) of serosanguinous fluid. During that time, the patient developed recurrent spiking high-grade fever along with increasing inflammatory markers. Therefore, cultures were drawn, and he was started on broad-spectrum antibiotics with blood cultures growing methicillin-sensitive *Staphylococcus aureus* (MSSA) and *Candida parapsilosis*. As his need for prolonged mechanical ventilation was expected, a tracheostomy was created early on in the course of his treatment. His treatment course was complicated by septic shock and neutropenic sepsis, requiring vasopressors, broad-spectrum antibacterials, and antifungals, along with granulocyte colony-stimulating factor (G-CSF). It was also complicated by a cholestatic pattern liver injury, which resolved with an improvement of his hemodynamics. In the SICU, the patient received three cycles of the VIP chemotherapy protocol and mechanical ventilation for 57 days. A follow-up chest CT scan showed a decrease in the size of the mass to 17 cm from 22 cm at its maximum. Serial alpha-fetoprotein tumor marker measurements showed progressively dropping levels. After transfer to the ward, the patient received a 4th cycle of VIP chemotherapy protocol with the last alpha-fetoprotein level of 177 IU/mL. He was then discharged with chest imaging done 32 days following the last VIP cycle, showing a tumor size of 13 cm in maximal diameter. He underwent a mediastinal mass resection through a median sternotomy incision with mass dissection from the aortic wall along with re-anastomosis of the right phrenic nerve. His postoperative course was complicated by lobar pneumonia, managed with intravenous antibiotics for three days, and then he was discharged. Histopathologic examination of the resected mass showed necrotic tissue with no evidence of malignancy. On follow-up 30 days after tumor resection, the chest, abdomen, and pelvis CT scans were remarkable for a clinically insignificant loculated pneumothorax. The alpha-fetoprotein level was 5.9 IU/mL, with otherwise unremarkable blood counts and metabolic and coagulation profiles. Currently, more than three years after his diagnosis, he is being followed up in the outpatient clinic with imaging and tumor markers, has no symptoms, and is fully functional.

## Discussion

The constellation of symptoms caused by mediastinal masses has been referred to by multiple names. Regardless of the terminology, the symptoms and signs of this syndrome are caused by a mediastinal mass causing compression of mediastinal structures within this confined space. When this compression involves the superior vena cava, leading to obstruction of venous blood flow within this central vein, the consequent congestive symptoms are termed SVC syndrome.

Primary mediastinal non-seminomatous GCTs are rare and have a poor prognosis [3]. Symptoms of a mediastinal mass are either caused by compression of the esophagus leading to dysphagia, compression of the SVC leading to congestion, or compression of the heart, lungs, or trachea leading to dyspnea, among other symptoms [5].

Described initially by Dr. Bittar, MMS is a right ventricular failure caused by vascular compression following positive pressure ventilation [6]. Mediastinal masses cause dynamic compression of the airways and major vascular structures within the mediastinum. Supine positioning, loss of spontaneous breathing due to deep sedation, and positive intrathoracic pressure exacerbate this effect, leading to a major compromise of hemodynamic and respiratory physiology.

Our patient had respiratory decompensation, which necessitated intubation and mechanical positive pressure ventilation. Though this was followed by respiratory and hemodynamic decompensation, it was manageable with IV fluid boluses and vasopressors, which augmented the cardiac preload upon which the hemodynamics of such patients depend. The suspicion of pulmonary embolism was high, considering his malignancy and the echocardiography findings. He likely developed cardiac arrest in the radiology department because of supine positioning combined with positive pressure ventilation. Of note, he did not experience any major decompensation while undergoing the previous imaging studies, likely because of a lack of positive pressure ventilation.

Circulatory collapse following the induction of anesthesia is a well-described complication. Multiple mechanisms have been presented, especially in a patient with poor cardiac and respiratory reserve. Our patient achieved a return of spontaneous circulation, likely due to manual ventilation as a part of his resuscitation, along with withholding sedation. The vascular and airway compression caused by mediastinal masses was previously intelligently described as dynamic, as the compression can be reversed, or at least ameliorated, with certain interventions [7].

Our initial goal was to relieve the compressing mass, which, in a non-seminomatous primary mediastinal GCT, is best achieved by cisplatin-based chemotherapy. This was not possible in the beginning, as the patient had active sepsis. Due to the chemotherapy-sensitive nature of these tumors, his respiratory condition improved dramatically with chemotherapy, eventually leading to weaning off mechanical ventilation and oxygen support.

In some institutions, it is accepted to start chemotherapy without tissue diagnosis in young adults with mediastinal masses and elevated levels of both AFP and beta-hCG [8]. As it is considered enough evidence for an extragonadal GCT. This practice is mostly used for critically ill patients with massive mediastinal masses, just like our patient. Unfortunately, we were not able to start his chemotherapeutic regimen before the treatment of his pneumonia. By the time his pneumonia was controlled, the histopathology result had been confirmed.

Although the surgical approach for patients with rising tumor markers has been controversial, several studies have concluded that resection of the remnant mass following chemotherapy gives the patient the best chance at long-term survival. This is compared to salvage chemotherapy, which adds little benefit and major toxicity to an already chemotherapy-resistant disease [9]. Kesler et al. recommended the surgical approach regardless of tumor marker levels following chemotherapy [10]. Their study also demonstrated the poor correlation between tumor marker levels and the presence of persistent non-seminomatous mediastinal GCT.

Our patient presented with a poor-risk malignancy that was complicated by cardiac arrest and a prolonged critical care course that encompassed pericardiocentesis, thoracentesis, chemotherapy, neutropenia, and multiple septic shock episodes. Lack of comorbidities, young age, prompt medical care, and the experience of the team likely contributed to his successful recovery.

## Conclusions

Mediastinal GCTs are challenging to manage. This sentence becomes more relevant when the tumor is complicated by mediastinal compression. Critically ill patients with large mediastinal masses and high tumor markers should be strongly considered for starting chemotherapy without attempting tissue diagnosis. In our opinion, more clear terminology may be the first necessary step toward clear guidance on the approach to such cases, which is definitely needed. Induction of anesthesia and initiating positive pressure ventilation may be the most critical steps in the management of patients with mediastinal masses, and these steps must be preceded by careful, thorough, and multidisciplinary evaluation. Finally, collaboration between institutions, societies, and countries may be the best option to collect enough data to generate confident recommendations.
